# Editorial for the special issue: The role of microorganisms in animal nutrition and metabolism

**DOI:** 10.3934/microbiol.2025038

**Published:** 2025-12-08

**Authors:** Uchenna Y. Anele, Ahmed E. Kholif

**Affiliations:** Department of Animal Sciences, North Carolina Agricultural and Technical State University, Greensboro, NC 24711, USA

Microorganisms are fundamental to animal nutrition and metabolism, and recent advances in microbiology, molecular techniques and systems science have revealed their extensive influence on livestock production. Across livestock species, diverse microbial communities regulate digestion, energy supply, immune function and even environmental performance. As global agriculture shifts toward more sustainable and efficient production systems, understanding and managing these microbial partners are crucial for the future of animal nutrition.

This special issue, “*The Role of Microorganisms in Animal Nutrition and Metabolism*,” brings together studies that highlight the breadth and practical relevance of microbial functions in modern animal agriculture. The articles explore microbial feed additives, functionally targeted probiotic strains, solid-state fermentation, advanced ensiling technologies and microbial fermentative pathways that influence host physiology. Collectively, these contributions show how targeted microbial interventions can improve nutrient availability, enhance animal health and support more environmentally responsible livestock production.

Microbial communities, such as bacteria, archaea, fungi, protozoa and viruses, perform key metabolic tasks that animals cannot accomplish alone. They ferment plant fibers, break down complex carbohydrates, synthesize vitamins and amino acids, and generate volatile fatty acids that sustain growth and productivity. In ruminants, the rumen acts as a natural bioreactor that converts lignocellulosic biomass into usable nutrients. In monogastric animals, the gut microbiota supports digestion, preserves gut integrity and shapes immune responses. When these ecosystems are disrupted, feed efficiency drops, disease susceptibility rises and metabolic functions shift.

New applications in feed science increasingly focus on guiding or enhancing microbial activity. Microbial feed additives, such as functional yeasts, bacterial probiotics and fungal supplements, are being developed to optimize fermentation, improve fiber degradation and reduce methane emissions. Fermentation-based feed processing technologies such as solid-state fermentation and advanced ensiling methods improve feed quality by favoring beneficial microorganisms and limiting spoilage. Functional probiotics, such as *Bacillus subtilis*, continue to show strong potential for improving nutrient absorption and supporting gut health and immunity. These developments align with global efforts to reduce antibiotic use and promote more sustainable livestock production systems.

The aim of this special issue is to create a focused platform for research at the intersection of microbiology, animal nutrition and metabolism. We seek to advance understanding of host–microbe interactions, encourage innovation in feed technologies and support interdisciplinary collaboration. Ultimately, we aim to highlight strategies that enhance productivity while reducing the environmental footprint of livestock production.

The studies in this special issue underscore the essential role of microorganisms in shaping the future of animal nutrition. Continued innovation and collaboration will help develop feeding strategies that support productive, healthy and environmentally sustainable livestock systems.

